# Health Economic Evaluation of a Controlled Lifestyle Intervention: The Healthy Lifestyle Community Program (Cohort 2; HLCP-2)

**DOI:** 10.3390/nu15245045

**Published:** 2023-12-08

**Authors:** Ragna-Marie Kranz, Carmen Kettler, Christian Koeder, Sarah Husain, Corinna Anand, Nora Schoch, Heike Englert

**Affiliations:** 1 Department of Food, Nutrition, Facilities, University of Applied Sciences Münster, 48149 Münster, Germany; carmen.kettler@fh-muenster.de (C.K.); christiankoeder@googlemail.com (C.K.); sarah.husain@fh-muenster.de (S.H.); corinna.anand@fh-muenster.de (C.A.); nora.schoch@fh-muenster.de (N.S.); englert@fh-muenster.de (H.E.); 2 Institute of Nutrition, Consumption and Health, Faculty of Natural Sciences, Paderborn University, 33098 Paderborn, Germany

**Keywords:** non-communicable diseases, cost analysis, health economics, prevention, community-based lifestyle intervention

## Abstract

Non-communicable diseases (NCD) are associated with high costs for healthcare systems. We evaluated changes in total costs, comprising direct and indirect costs, due to a 24-month non-randomized, controlled lifestyle intervention trial with six measurement time points aiming to improve the risk profile for NCDs. Overall, 187 individuals from the general population aged ≥18 years were assigned to either the intervention group (IG; *n* = 112), receiving a 10-week intensive lifestyle intervention focusing on a healthy, plant-based diet; physical activity; stress management; and community support, followed by a 22-month follow-up phase including monthly seminars, or a control group (CG; *n* = 75) without intervention. The complete data sets of 118 participants (IG: *n* = 79; CG: *n* = 39) were analyzed. At baseline, total costs per person amounted to 67.80 ± 69.17 EUR in the IG and 48.73 ± 54.41 EUR in the CG per week. The reduction in total costs was significantly greater in the IG compared to the CG after 10 weeks (*p* = 0.012) and 6 months (*p* = 0.004), whereas direct costs differed significantly after 10 weeks (*p* = 0.017), 6 months (*p* = 0.041) and 12 months (*p* = 0.012) between the groups. The HLCP-2 was able to reduce health-related economic costs, primarily due to the reduction in direct costs.

## 1. Introduction

Diseases pose a major challenge to the healthcare system due to the enormously high costs associated with them. Of particular relevance in terms of healthcare costs are chronic diseases and non-communicable diseases (NCDs) [1]. The prevalence of NCDs, such as obesity, cardiovascular disease, diabetes mellitus type 2, cancer and mental illnesses, is increasing worldwide [2]. Consequently, the expenses for the treatment and aftercare of the diseases are also rising, and these costs constitute a heavy burden on health systems [1,3,4]. In Germany, cardiovascular diseases cause the main part of health expenditure, yet systematic data on the health economic impact of all NCDs have not yet been collected in Germany [5]. However, due to their increasing relevance and actuality, the Robert Koch Institute (RKI) intends to establish a national NCD surveillance scheme in the future [6].

To address these challenges, primary and secondary prevention of lifestyle-related conditions by reducing risk factors constitute an important strategy [7,8,9]. Long-term lifestyle changes, such as having a nutrient-rich, plant-based diet; regular physical activity; maintenance of body weight; and the cessation of smoking can improve clinical parameters and subjective perceptions and are, therefore, likely to reduce costs for healthcare systems [10,11,12]. For this purpose, both healthy individuals and people with an increased risk profile for NCDs and/or associated diagnosis should be addressed [13].

Published data from the Healthy Lifestyle Community Program (HLCP-1,HLCP-2 and HLCP-3), an intensive lifestyle intervention with a community-based approach, have shown that the program can result in metabolic and anthropometric improvements [14,15,16,17]. For body weight, the primary outcome parameter, we had hypothesized that the lifestyle intervention would lead to a significant reduction within the intervention group (IG) compared to the control group (CG) [17]. To ensure that the program will not only improve the health of individuals but also result in benefits for the healthcare system, it is essential to examine the impact of this community-based lifestyle intervention on healthcare costs. For this purpose, direct healthcare costs, including costs for prevention, treatment or medications, are distinguished from indirect costs, which describe the loss of labor or income due to disease [18].

To the best of our knowledge, there are no known health economic evaluations of community-based lifestyle interventions in real-world settings with a focus on NCDs that examine the impact of lifestyle interventions on participants’ total healthcare costs from a societal perspective. Most studies to date have focused on a cost-effectiveness analysis, relating the costs of the program to the effects achieved or targeting specific disease patterns and separate lifestyle factors [19,20,21,22,23,24,25,26].

Therefore, we conducted a health economic analysis of the HLCP-2 by surveying total healthcare costs, i.e., participant-associated direct and indirect costs during the course of this study, as a secondary outcome of this study. In this regard, the hypothesis of this study was that the intervention would significantly reduce total, direct and indirect costs in the IG during the study period compared to baseline, both within the IG and compared to the CG.

## 2. Materials and Methods

### 2.1. Study Design

A non-randomized, controlled intervention trial with a community-based approach was used to measure the effect of lifestyle changes on NCD risk parameters. Data were collected at 6 time points over a 2-year period: baseline (t_0_) and after 10 weeks (t_1_), 6 months (t_2_), 12 months (t_3_), 18 months (t_4_) and 24 months (t_5_). Due to organizational reasons, the CG started and finished 6 months later than the IG. The time interval of follow-ups was the same in both groups. At the last time point (t_5_), health check-ups could not be conducted in person due to the COVID-19 pandemic and contact restrictions. However, all parameters that could be collected via questionnaires were collected, as participants sent the documents via post. The IG participated in the lifestyle intervention program; the CG received no intervention. The participants of both groups were informed about their health check-up results for ethical reasons. This study was conducted in accordance with the Declaration of Helsinki. The study protocol was approved by the ethics committees of the Medical Association of Westphalia–Lippe and the University of Muenster (Muenster, Germany; reference: 2018-171-f-S; approved 4 April 2018), and the trial was registered in the German Clinical Trial Register (DRKS; reference: DRKS00018775; www.drks.de, accessed on 7 December 2023).

### 2.2. Study Population

Participants were recruited in two different rural municipalities in north-west Germany to separate the groups based on location. The two municipalities were comparable in terms of socio-demographic and cultural characteristics and located in the same region, although the intervention municipality had around twice as many inhabitants as the control municipality. All individuals from the general population who were at least 18 years of age and had the physical and mental ability to take part in this study were eligible to participate in this study. Local stakeholders were involved in the recruitment of participants. A cooperative health market was conducted in the intervention municipality (February 2018) with the aim of recruiting participants for this study. Participants in the CG were mainly recruited at a local event (September 2018). Interested citizens were able to register for the IG or CG during these events, depending on where they lived. Distributing flyers and publishing newspaper articles were also used to recruit participants in both municipalities. Due to the complex population-based public health approach, in which the intervention was conducted in a real-world context to permit the examination of authentic behavior, the randomization of participants was not feasible (as described previously [15]) [27]. The blinding of participants or instructors to group allocation was not possible, so only laboratory staff were blinded (as described previously [15]). All subjects gave their written informed consent prior to inclusion in this study.

### 2.3. Measures

For data collection, participants completed 2 different questionnaires at all 6 measurement time points: 1 to record socioeconomic data and 1 to assess health economic parameters. In the questionnaires, participants reported direct costs due to regular medication use, physician´s visits, check-ups and treatments, therapeutic appliances and remedies, inpatient and outpatient visits, ambulance services and care services retrospectively for the time period of the last 10 weeks.

In a pilot study with 61 patients, the comprehensibility and accuracy of the health economic variables in the questionnaire were tested and verified, using their physicians’ data for comparison [26]. If there was any incomprehensibility, the wording of each question was changed accordingly. The questionnaires derived from this pilot study were already successfully applied in an open-label, 2-arm parallel-group randomized controlled trial of cost calculation in Germany [26]. For the HLCP-2, these questionnaires were updated and slightly adapted to fit this study’s purpose and the target group.

A clear allocation of the costs proved to be difficult, as there is no consistent procedure in Germany. For this reason, Scholz et al. (2020) provided a suggestion for a standardized cost allocation for Germany, on which our approach for cost calculation is based (Table 1) [28]. Thereby, a bottom-up approach from a societal perspective was taken to consider the resource use of all stakeholders (including health insurers, patients and employers). Physicians’ costs were corrected for the flat-rate payment that is currently used in the German healthcare system, i.e., the cost of treating patients is calculated, and the provider receives payment that covers all diagnostic and curative services related to the diseases, regardless of the actual scope and duration of treatment [29]. Indirect costs were calculated in terms of the time of incapacity to work and early retirement.

To precisely calculate costs, participants indicated their health insurance affiliation. Costs for the statutory health insurance fee were based on the physician’s fee scale for statutory insurance in Germany (EBM, “Einheitlicher Bewertungsmaßstab”), and costs for private insurance were based on the physician’s fee scale for private insurance in Germany (GoÄ, “Gebührenordnung für Ärzte”) [28]. For better comparability, the calculated expenses of each cost component were always calculated on a weekly basis. In addition to total costs, direct and indirect costs were separately analyzed.

In addition, data on health behavior, quality of life and dietary intake (semiquantitative 3-day food protocols), as well as blood parameters (e.g., blood lipids, fasting glucose and HbA1c), anthropometric parameters (e.g., body weight and waist circumference) and vital parameters (resting heart rate, and blood pressure), were collected during all health check-ups, except for t_5_ in the CG. Blood, anthropometric and vital parameters were assessed in the fasted state. For accuracy, data entry was performed twice.

### 2.4. Intervention

The intervention consisted of two phases: a 10-week intensive phase and a less intensive, 22-month alumni phase [30]. The first phase included 14 consecutive seminars with practical units, each lasting two hours and offered twice a week. Interactive workshops for smaller groups (20 participants) were also offered in this phase. The main focus of the intervention was on four areas of lifestyle change: a healthy, predominantly plant-based diet; physical activity; stress management; and social support [31,32,33,34].

Dietary recommendations of the HLCP-2 included consuming more healthy plant-based foods, like vegetables, fruits, legumes, whole grains, seeds, nuts and healthy oils. In addition, participants were encouraged to reduce the intake of less healthy foods, such as meat, high-fat dairy products, highly processed foods and added sugar and salt [34]. At least 30 min of physical activity per day, reducing sitting time, joining physical activity support groups such as walking groups, and choosing one’s own pace for physical activity were also recommended. In terms of stress management, recommendations included integrating relaxation routines and breaks into daily routines, attempting stress reduction courses, practicing mindfulness and establishing individual coping strategies for stress reduction. Concerning community support, participants were encouraged to support each other’s healthy lifestyles, form “support groups” (e.g., for cooking, eating together or walking) and ask their friends and family to support their healthy lifestyles.

A healthy lifestyle handbook, an information sheet with an overview of the key lifestyle recommendations and a recipe booklet were handed out to the participants [35]. Additionally, each participant was offered 2 health coaching sessions (1-on-1). Individual goals were set at baseline, and the reaching of these goals was discussed after the 10-week intensive phase. The less intensive, 22-month alumni phase included newsletters and monthly seminars to support long-term behavior change.

### 2.5. Analysis

The sample size was calculated on the basis of the change in body weight, which was the primary outcome parameter of this study. The calculation was conducted using data from a pilot study with a prototype version of the lifestyle program (as described previously [15,17]). Eligible participants were included in the present secondary analyses. All analyses were based on a pre-defined statistical analysis plan and performed on complete data sets (t_0_–t_5_). Outliers, defined as values < 25% quantile − 1.5 × interquartile range (IQR) and >75% quantile + 1.5 × IQR, were excluded from the statistical analysis. Descriptive statistics for quantitative variables are reported as means ± standard derivation (SD). Categorial data are expressed as absolute numbers and percentages (%). The Shapiro–Wilk test was used to assess the data for non-normality, and *p* < 0.05 was defined as describing a non-normal distribution.

For between-group comparisons, Fisher’s exact test was used for categorical variables, while an independent *t*-test was used for normally distributed continuous variables and the Mann–Whitney-U test was used for non-normally distributed continuous variables. To evaluate within-group changes, a paired *t*-test was used for normally distributed data and the Wilcoxon signed-rank test was used for non-normally distributed data. All tests performed were two sided.

Multiple linear regression models were used to compare the intervention and control groups regarding changes in total costs (adjusted for covariates). Confounders were identified and considered as potential covariates in the multiple regression models. Firstly, univariate analyses were conducted with the following variables to identify potential covariates: sociodemographic data (sex, age, marital status and education level), blood parameters (total cholesterol, HDL cholesterol, LDL cholesterol, triglycerides, fasting blood glucose, HbA1c), vital signs (systolic and diastolic blood pressure and resting heart rate), body weight, waist circumference and BMI, total costs at baseline, smoking status, assignment to IG or CG, alcohol consumption, sleep quality, diagnosed disease and regular medication use were examined. Secondly, AIC-based forward selection was performed to find final models that were statistically significant (general linear F-test: *p* ≤ 0.05) and had the highest corrected R^2^ and the fewest covariates. Residuals were inspected to check for normality. For all analyses, results were considered significant at *p* < 0.05. SPSS version 27 for Windows was used (SPSS Inc., Armonk, NY, USA).

## 3. Results

**Figure 1:**
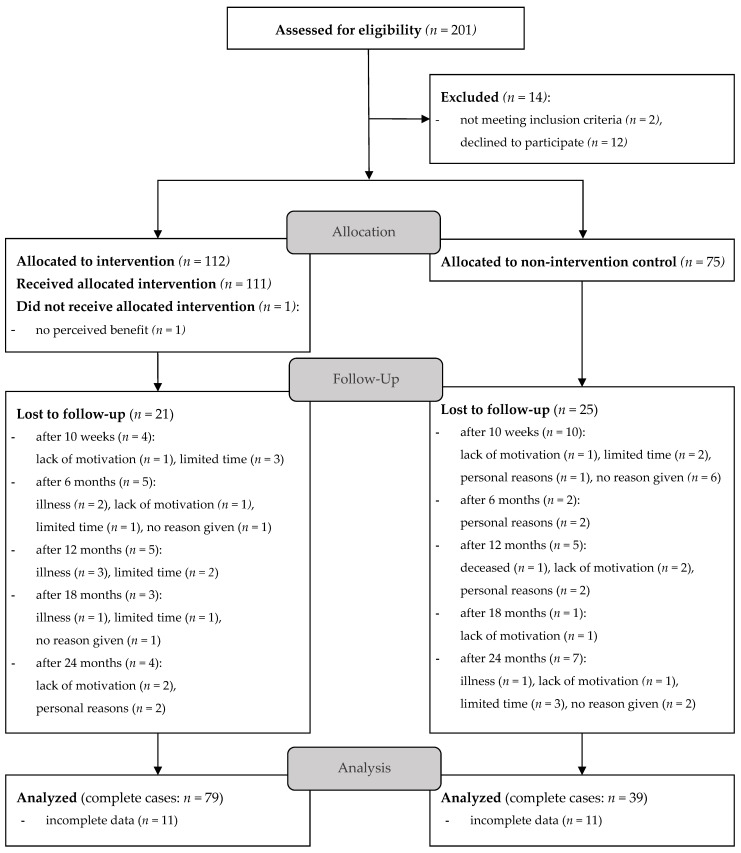
CONSORT participants’ flow diagram; participants categorized as “lost to follow-up” withdrew from this study for the given reason.

Figure 1 shows the participants´ flow through the study from enrollment to analysis. In total, 118 participants (IG: *n* = 79; CG: *n* = 39) were considered during the analysis due to their being complete data sets for all 6 measurement time points (t_0_–t_5_).

### 3.1. Baseline Characteristics

The baseline characteristics of the IG and CG are shown in Table 2. The demographic characteristics and clinical parameters of both study arms were comparable (*p* > 0.05). The study participants were middle-aged (age at baseline, IG: 59.2 ± 7.8 years; CG: 56.4 ± 9.2 years), overweight (BMI, IG: 27.4 ± 5.3 kg/m^2^; CG: 28.1 ± 5.7 kg/m^2^) and had at least one long-term health condition, e.g., hypertension, diabetes type 2 or a musculoskeletal disorder (IG: 81.0%; CG: 74.4%).

Significant differences between participants in the IG and the CG were only observed regarding age (*p* = 0.031) and education level (*p* = 0.019), whereby participants in the IG were older and had a higher education level. The majority of participants in both study groups were members of the statutory health insurance scheme (IG: 82.3%; CG: 89.7%; *p* = 0.092).

### 3.2. Calculation of Costs

Table 3 presents total, indirect and direct costs per person per week (in EUR) for the IG and CG at the first measurement time point (t_0_) and changes in costs after 10 weeks (t_1_), 6 (t_2_), 12 (t_3_), 18 (t_4_) and 24 (t_5_) months (compared to baseline). At baseline, health economic costs totaled 67.80 ± 69.17 EUR in the IG and 48.73 ± 54.41 EUR in the CG. Total costs and direct costs were comparable between the two study groups (total costs: *p* = 0.102; direct costs: *p* = 0.053), while indirect costs differed significantly between the IG and CG (*p* = 0.014). The changes in total costs compared to baseline differed significantly between the study groups after 10 weeks (*p* = 0.012) and 6 months (*p* = 0.004). Total costs decreased significantly within the IG at all measurement time points compared to baseline (t_0_–t_1_: *p* < 0.001; t_0_–t_2_: *p* < 0.001; t_0_–t_3_: *p* < 0.001; t_0_–t_4_: *p* < 0.001; t_0_–t_5_: *p* = 0.003). Within the CG, total costs tended to decrease from 10 weeks onwards, and this variable was significant at 18 months (*p* = 0.015).

Indirect costs (early retirement and time of incapacity to work) accounted for a smaller share of total costs than direct costs. The change in direct costs compared to baseline differed significantly between the IG and CG after 10 weeks (*p* = 0.017), 6 months (*p* = 0.041) and 12 months (*p* = 0.012). As with total costs, direct costs (IG at t_0_: 47.84 ± 42.80 EUR; GC at t_0_: 33.21 ± 33.65 EUR) decreased significantly within the IG at all measurement time points compared to baseline (t_0_–t_1_: *p* < 0.001; t_0_–t_2_: *p* < 0.001; t_0_–t_3_: *p* < 0.001; t_0_–t_4_: *p* < 0.001; t_0_–t_5_: *p* = 0.003). No significant changes in direct costs were identified within the CG.

Changes in indirect costs from baseline differed significantly between the two study groups after 10 weeks (*p* = 0.021) and 6 months (*p* = 0.044). At baseline, indirect costs in the IG were 11.39 ± 29.13 EUR per week, and these costs decreased at all measurement time points compared to baseline (t_0_–t_1_: *p* = 0.005; t_0_–t_2_: *p* = 0.007; t_0_–t_3_: *p* = 0.026; t_0_–t_4_: *p* = 0.017; t_0_–t_5_: *p* = 0.027). Since outliers were excluded from the analysis, the CG did not have associated indirect costs at any measurement time point.

To adjust for group differences arising from potential confounders, multiple linear regression (MLR) analyses were performed (Table 4). The change in total health economic costs (compared to baseline) was considered as a dependent or target variable. The variable differed significantly between the two study groups after 10 weeks (*p* = 0.005) and 6 months (*p* < 0.001). Beyond that point, costs at baseline had a significant impact on the reduction in total costs in all final models (10 weeks and 6, 12, 18 and 24 months: *p* < 0.001), where higher baseline costs meant higher cost reduction. After 18 months, a higher diastolic blood pressure at baseline was associated with a greater reduction in health economic costs (*p* = 0.029). Other parameters, i.e., blood, anthropometric and vital parameters, marital status, education level, quality of sleep and alcohol consumption, had no recognizable influence on the change in total costs.

## 4. Discussion

This health economic evaluation shows that the community-based lifestyle intervention was able to reduce total costs over the 24-month course of this study compared to baseline. Compared to the CG, total costs were significantly reduced at t_1_ and t_2_ in the IG. In addition to the group allocation at t_1_ and t_2_, the total costs at baseline were identified as relevant determinants at all time points in the MLR. Within the IG, total costs were significantly reduced at all time points compared to baseline. The largest cost reduction was 73% (t_0_–t_1_), reducing total costs by −49.73 ± 65.59 EUR per person per week. Consequently, the HLCP-2 successfully contributed to a reduction in total costs. However, it should be considered that the implementation of the lifestyle intervention also incurred costs. A calculation of the intervention costs is difficult for the HLCP-2, as it is a community-based study approach that partly relied on the (voluntary) involvement of local stakeholders. In addition, costs incurred within the context of scientific studies may not be considered representative due to the inclusion of costs for long follow-up assessments and additional diagnostic procedures, as well as costs for planning and implementing interventions according to scientific standards that are incurred and other cost components, e.g., for concomitant medications, are not sufficiently taken into account [26]. Nevertheless, conducting a cost-effectiveness analysis, considering the actual intervention costs, could contribute to a better overview of the potential cost savings.

The significant reduction in total costs after 18 months within the CG (−22.43 ± 46.82 EUR; *p* = 0.015) may be attributed to the COVID-19 pandemic. At this time, the first lockdown occurred in Germany, and medical care was strongly reduced, meaning that necessary treatments for non-urgent illnesses were cancelled or postponed and, among other things, fewer hospital stays and treatments took place [36]. In addition, participants in the CG were given their health check results, which may have influenced their lifestyles and, thus, costs.

Regarding the HLCP-2, the cost reduction was primarily attributed to direct costs (including costs of medications, physician visits, treatments, therapeutic appliances and remedies). Direct costs were significantly improved in the IG intra-group comparison at all time points and in the CG inter-group comparison at all time points, except for the last two time points in the baseline comparison. Our results could be attributed to an improvement in clinical parameters, such as body weight, BMI, waist circumference, resting heart rate, blood lipids and fasting glucose, which changed significantly more in the IG than in the CG after one year compared to baseline due to a change in lifestyle [17]. These results are consistent with the literature results showing that improving lifestyle and NCD risk factors led to reductions in healthcare costs [7,11,37]. A separate analysis of the costs of selected NCD medications was conducted and showed a significantly higher reduction in the costs of NCD medications after 6, 12, 18 and 24 months compared to baseline in the between-group comparisons, with the greatest effect made on antihypertensive drugs [38].

The indirect costs could only be improved to a small extent by the 2-year HLCP-2 intervention (t_1_ and t_2_) or the changes were not significant (t_3_, t_4_, and t_5_; comparison of IG and CG), which can primarily be related to the exclusion of outliers in indirect costs. Outliers were excluded from the analysis because several cost parameters, especially costs for early retirement, hospital and rehabilitation stays, were extremely high, thus distorting the mean values.

These outliers were largely related to parameters that cannot be influenced by lifestyle changes in the short term. For example, early retirement is associated with chronic diseases, unhealthy lifestyles behavior and poor health [39,40], and these cannot easily be eliminated through short-term behavioral changes and the accompanying improvements in clinical parameters, which could reduce indirect costs. Nevertheless, the observed trend in the reduction in indirect costs within the IG could be attributed to a reduction in days of incapacity to work as a result of better general health status during the course of the study.

A German randomized controlled trial by Müller-Nordhorn et al. (2008) that used the same health economic questionnaires found that indirect costs (56%) were proportionately higher than direct costs (44%), with disease-related early retirement responsible for the highest costs (42%) in patients with hypercholesterolemia, which differs from our study results [26]. However, it should to be noted that the study only focused on patients with a diagnosed hypercholesterolemia, the cost calculation in that study was based on different principles (compared to the present study) for cost allocation and medical costs may have changed in recent years [26].

Although there are no directly comparable studies, there are several studies that address the relationships between lifestyle factors and health economic issues related to NCDs and emphasize the cost-saving potential of these interventions [20]. The relationship between lifestyle factors and health-related costs was also identified in a study by Sarria-Santamera et al. (2022), who analyzed the impact of physical activity in people with diabetes on direct and indirect costs in Spain. They showed that even a minimal amount of physical activity was associated with substantial reductions in direct and indirect costs [37]. Physical activity was also a focus within the HLCP-2 intervention, with recommendations to increase daily physical activity and achieve moderate improvements made individually. In addition, dietary recommendations were a key component of the HLCP-2, which were also focused on in the cost-effectiveness study of Dorhout et al. (2021), who found that their intervention, a combined diet and resistance training intervention among older adults in the Netherlands, had an 82.4% probability of being cost effective [22]. In this Dutch study, direct healthcare costs during the 24-week study period were also higher in the CG compared to the IG, as shown in particular by lower costs for physiotherapy, home care and medication [22]. Similarly, several studies of type 2 diabetes prevention for high-risk individuals have been shown to be cost effective or cost saving [20,24]. Therefore, group-based programs proved to be more cost-effective than those that used individual sessions [24]. The HLCP-2 intervention was offered in group sessions, except for the 1-on-1 health coaching sessions, saving staff and time resources while promoting social support.

A strength of the present study is the use of a non-intervention control group, the assessment of a large variety of parameters and the complex real-world setting. All health economic parameters were collected at all six measurement points, which permits an evaluation of the costs over a period of 2 years. By means of the specific collection of health economic parameters, individual and specific cost allocation from a societal perspective was possible. 

The main limitation of this study is that the CG started with a delay of 6 months (same follow-up duration) compared to the IG (IG: April 2018; CG: October 2018). While both groups were comparable at baseline, except for age and education level, and we adjusted for potential confounders in the multiple linear regression analysis, some bias, such as selection bias, may have remained. Additionally, the sample size was rather small, and some time points (IG: t_5;_ CG: t_4_ and t_5_) occurred after the start of the COVID-19 pandemic, which may have influenced lifestyle behavior, clinical parameters and health costs [41]. Furthermore, health economic analyses are very heterogeneous depending on the study design, perspective, country, topicality, healthcare and financing system and are, therefore, difficult to compare [25,42]. For example, in our analysis, physicians’ costs were corrected for the flat-rate payment. This is a billing system that is currently established in Germany, but its benefits are highly debated [29]. Therefore, our results can most easily be compared in the context of the German healthcare system and the associated costs and billing systems.

## 5. Conclusions

In summary, the HLCP-2 was mainly able to reduce total costs through a reduction in direct costs because of healthy lifestyle changes. Within the IG, total costs could be significantly reduced over the 2-year period (compared to baseline, as well as the CG, after 10 weeks and 6 months), with the greatest cost reduction noted after the 10-week intensive phase. Nevertheless, healthcare systems currently have not systematically incorporated lifestyle treatments into clinical practice and often do not cover the costs of nutrition services [22]. To make the healthcare system and policy-makers aware of the benefits of lifestyle interventions, large-scale health economic evaluations of lifestyle intervention studies should be conducted in the future. Apart from the content level of a holistic lifestyle intervention, we should also address how evidence-based knowledge can be implemented and established in different settings in the long term. The results of the HLCP-2 study may sensitize decision-makers to the potential of community-based lifestyle interventions and encourage them to prioritize and allocate resources to the primary and secondary prevention of NCDs in real-life settings.

## Figures and Tables

**Table 1:** Data sources for the cost factors surveyed in the questionnaires [28].

	Segment	Information Base
Direct costs	Medications	Lauer-Taxe^®^
	Ambulatory physician sector	Report on the results of the 2019 fee distribution
	Treatments	Statutory health insurance: EBM; private health insurance: GoÄ
	Therapeutic appliances	Reference amounts of the National Association of Statutory Health Insurance Funds; average prices of an online medical supply store
	Remedies	Remedies prices of the National Association of Statutory Health Insurance Funds
	Inpatient visit	Report on the results of the 2019 fee distribution
	Outpatient visits	Hospital: hospital statistics based on flat rates per case (DRG-statistics); rehabilitation clinic, when employed: rehabilitation report 2019 of the German pension insurance; rehabilitation clinic, when unemployed: statistics of the Federal Ministry of Health
	Ambulance service	Local statutes of the municipality
	Care service	German Code of Social Law
Indirect costs	Time of incapacity to work	Statistical yearbook 2019
	Early retirement	Statistical yearbook 2019

**Table 2:** Baseline characteristics by study groups (complete cases).

Variable	Intervention Group (*n* = 79)	Control Group (*n* = 39)	*p*-Value
**Age, years: mean ± SD**	59.2 ± 7.8	56.4 ± 9.2	0.031 ^c^
**Sex, n (%)**			0.402 ^a^
Male	23 (29.1)	15 (38.5)	
Female	56 (70.9)	24 (61.5)	
**Body weight, kg: mean ± SD**	80.7 ± 19.0	83.9 ± 18.1	0.260 ^c^
**BMI, kg/m^2^: mean ± SD**	27.4 ± 5.3	28.1 ± 5.7	0.542 ^c^
**Waist circumference, cm: mean ± SD**	97.5 ± 15.1	96.8 ± 14.8	0.975 ^c^
**Education level, n (%)**			0.019 ^a^
Lower secondary school	14 (17.7)	14 (35.9)	
Secondary school	36 (45.6)	12 (30.8)	
University entrance qualification	14 (17.7)	11 (28.2)	
University degree	15 (19)	2 (5.1)	
**Marital status, n (%)**			0.547 ^a^
Married	63 (79.7)	35 (89.7)	
Partner, unmarried	5 (6.3)	1 (2.6)	
Single (not widowed)	7 (8.9)	1 (2.6)	
Single (widowed)	4 (5.1)	2 (5.1)	
**Blood pressure: mean ± SD**			
Systolic blood pressure, mm Hg	133.3 ± 15.0	134.9 ± 14.5	0.585 ^b^
Diastolic blood pressure, mm Hg	81.3 ± 9.0	80.7 ± 9.3	0.636 ^c^
**Blood parameters: mean ± SD**			
Total cholesterol, mg/dL	209.2 ± 38.0	205.9 ± 44.8	0.677 ^b^
HDL cholesterol, mg/dL	66.6 ± 18.9	62.1 ± 17.9	0.226 ^c^
LDL cholesterol, mg/dL	134.1 ± 36.2	138.5 ± 41.5	0.555 ^b^
Triglycerides, mg/dL	104.6 ± 52.7	112.6 ± 49.3	0.292 ^c^
Fasting glucose, mg/dL	98.3 ± 11.5	102.0 ± 12.5	0.352 ^c^
HbA1c, %	5.4 ± 0.5	5.5 ± 0.4	0.691 ^c^
**Health insurance, n (%)**			0.092 ^a^
Statutory health insurance	65 (82.3)	35 (89.7)	
Private health insurance	13 (16.5)	2 (5.1)	
Other	1 (1.3)	2 (5.1)	
**Diagnosis of diseases, n (%)**	64 (81.0)	29 (74.4)	0.474 ^a^
**Medication use (regularly), n (%)**	57 (72.2)	23 (59.0)	0.208 ^a^
**Employment, n (%)**			0.129 ^a^
Employed	50 (63.3)	29 (74.4)	
Pensioner	25 (31.6)	6 (15.4)	
Unemployed	0 (0.0)	0 (0.0)	
Housewife/househusband	4 (5.1)	4 (10.3)	

SD: standard deviation; BMI: body mass index; ^a^ Fisher’s exact test; ^b^ Independent *t*-test; ^c^ Mann–Whitney U test.

**Table 3:** Health economic costs (in EUR) per week during the study course (t_0_–t_5_) for the intervention (IG) and control (CG) groups.

				Change after									
			Costs at Baseline	10 Weeks	*p*-Value #	6 Months	*p*- Value #	12 Months	*p*- Value #	18 Months	*p*- Value #	24 Months	*p*- Value #
**Total costs ^1^**	IG	mean ± SD	67.80 ± 69.17	−49.73 *** ± 65.59	0.012	−40.91 *** ± 60.00	0.004	−28.83 *** ± 60.57	0.259	−39.71 *** ± 62.50	0.240	−22.92 ** ± 85.67	0.305
	CG	mean ± SD	48.73 ± 54.41	−18.40 ± 53.77		0.39 ± 70.57		−23.73 ± 54.60		−22.43 * ± 46.82		−18.25 ± 53.48	
**Direct costs ^2^**	IG	mean ± SD	47.84 ± 42.80	−24.92 *** ± 34.90	0.017	−20.44 *** ± 33.83	0.041	−21.83 *** ± 33.64	0.012	−21.70 *** ± 36.06	0.070	−18.66 *** ± 43.19	0.243
	CG	mean ± SD	33.21 ± 33.65	−5.97 ± 23.76		−4.98 ± 26.09		−5.66 ± 31.11		−7.39 ± 32.05		−8.91 ± 30.99	
**Indirect costs ^3^**	IG	mean ± SD	11.39 ± 29.13	−11.06 ** ± 29.28	0.021	−10.38 ** ± 28.85	0.044	−6.23 * ± 19.80	0.057	−6.96 * ± 22.68	0.059	−7.33 * ± 24.00	0.058
	CG	mean ± SD	0.00 ± 0.00	0.00 ± 0.00		0.00 ± 0.00		0.00 ± 0.00		0.00 ± 0.00		0.00 ± 0.00	

Wilcoxon signed-rank test for within-group differences with * *p* < 0.05. ** *p* < 0.01. *** *p* < 0.001 for within-group comparisons at baseline; Mann–Whitney-U test for between-group differences; # *p*-value for between-group comparisons of the change in costs (reference: t_0_). IG: intervention group; CG: control group; SD: standard derivation. ^1^ IG: t_0_: *n* = 69; t_1_–t_0_: *n* = 66; t_2_–t_0_: *n* = 62; t_3_–t_0_: *n* = 61; t_4_–t_0_: *n* = 62; t_5_–t_0_: *n* = 63. CG: t_0_: *n* = 37; t_1_–t_0_: *n* = 34; t_2_–t_0_: *n* = 36; t_3_–t_0_: *n* = 35; t_4_–t_0_: *n* = 32; t_5_–t_0_: *n* = 34. ^2^ IG: t_0_: *n* = 75; t_1_–t_0_: *n* = 69; t_2_–t_0_: *n* = 64; t_3_–t_0_: *n* = 69; t_4_–t_0_: *n* = 66; t_5_–t_0_: *n* = 70. CG: t_0_: *n* = 38; t_1_–t_0_: *n* = 35; t_2_–t_0_: *n* = 38; t_3_–t_0_: *n* = 37; t_4_–t_0_: *n* = 35; t_5_–t_0_: *n* = 36. ^3^ IG: t_0_: *n* = 66; t_1_–t_0_: *n* = 64; t_2_–t_0_: *n* = 63; t_3_–t_0_: *n* = 56; t_4_–t_0_: *n* = 61; t_5_–t_0_: *n* = 55. CG: t_0_: *n* = 33; t_1_–t_0_: *n* = 31; t_2_–t_0_: *n* = 26; t_3_–t_0_: *n* = 32; t_4_–t_0_: *n* = 29; t_5_–t_0_: *n* = 31.

**Table 4:** Multiple linear regression models for change in total costs per week (in EUR) after 10 weeks, 6, 12, 18 and 24 months compared to baseline.

	ß	SE	*p*-Value
**10 weeks ^1^**			
constant (ß_0_)	9.400	3.566	0.010
group (ref. intervention)	13.697	4.733	0.005
total costs at baseline	−0.875	0.034	<0.001
**6 months ^2^**			
constant (ß_0_)	15.275	5.710	0.009
group (ref. intervention)	28.236	7.282	<0.001
total costs at baseline	−0.896	0.058	<0.001
**12 months ^3^**			
constant (ß_0_)	20.189	5.153	<0.001
group (ref. intervention)	−3.489	6.691	0.603
total costs at baseline	−0.847	0.056	<0.001
**18 months ^4^**			
constant (ß_0_)	−33.271	21.496	0.125
group (ref. intervention)	4.827	5.121	0.348
diastolic blood pressure	0.587	0.265	0.029
total costs at baseline	−0.873	0.040	<0.001
**24 months ^5^**			
constant (ß_0_)	33.598	8.566	<0.001
group (ref. intervention)	−9.742	11.220	0.387
total costs at baseline	−0.880	0.085	<0.001

Dependent variable: change in total costs (compared to baseline). ^1^ corr. R^2^ = 0.877. FS, F-Test: *p* < 0.001; ^2^ corr. R^2^ = 0.733. FS, F-Test: *p* < 0.001. ^3^ corr. R^2^ = 0.708. FS, F-Test: *p* < 0.001; ^4^ corr. R^2^ = 0.841. FS, F-Test: *p* < 0.001. ^5^ corr. R^2^ = 0.522. FS, F-Test: *p* < 0.001. All residuals are normally distributed. SE: standard error; ref.: reference group, FS: forward selection; corr. R^2^: corrected R^2^; F-Test: general linear F-Test.

## Data Availability

The data presented in this study are available on request from the corresponding author (R.-M.K.).
